# Current knowledge of burn injury first aid practices and applied traditional remedies: a nationwide survey

**DOI:** 10.1186/s41038-016-0063-7

**Published:** 2016-11-02

**Authors:** Abdullah E. Kattan, Feras AlShomer, Abdulaziz K. Alhujayri, Abdullah Addar, Albaraa Aljerian

**Affiliations:** Plastic and Reconstructive Surgery Unit, King Khalid University Hospital, College of Medicine, King Saud University, 37 P. O. Box 7805, Riyadh, 11472 Kingdom of Saudi Arabia

**Keywords:** Burn, First aid, Practices, Traditional remedies

## Abstract

**Background:**

Burn first aid awareness has been shown to reduce morbidity and mortality. We present a report on the knowledge and practices of the Saudi population with regard to burn first aid and the application of traditional remedies.

**Methods:**

An internet-based survey was conducted to assess the public’s knowledge on first aid practices and home remedies applied for burn injuries among Saudi adults.

**Results:**

A total of 2758 individuals responded to the survey. There were 1178 (42.7 %) respondents who had previously received burn first aid information. One thousand five hundred fifty respondents had a history of burn exposure in which burn injury first aid was applied as follows: 1118 (72.1 %) removed clothing and accessories from the injured area; water was applied by 990 (63.9 %); among those who applied water, 877 (88.6 %) applied cold water; and only 57 (5.8 %) did so for more than 15 min. Wrapping the burn area was performed by 526 (33.9 %), and 985 (63.5 %) sought medical assistance. When it comes to traditional remedies, 2134 (77.4 %) knew of and/or implemented these remedies as first aid or to treat burns. Honey and toothpaste were the commonest among these remedies with 1491 (69.9 %) and 1147 (53.7 %), respectively. This was associated with female gender (*r* = 0.87, *P* < 0.001), younger age group (19–25 years) (*r* = 0.077, *P* < 0.001), from central region (*r* = 0.012, *P* < 0.001), and university graduate (*r* = 0.05, *P* = 0.002). Nearly half of those who knew of traditional remedies did not have previous knowledge of burn first aid.

**Conclusions:**

Proper burn first aid is a simple, cheap, and accessible means of managing burns initially. Although the majority of the respondents were university graduates (51.1 %), knowledge and implementation of burn first aid was very poor. Major healthcare agencies should review and promote a consistent guideline for burn first aid in an effort to tackle and minimize the effect of this grave injury.

## Background

Burns are potentially devastating injuries with many consequences ranging from physical, functional, and occupational to cosmetic and psychosocial damage. Proper knowledge about burn first aid minimizes the overall impact of the injury. This study was conducted in an effort to assess the level of awareness of the Saudi population regarding burn first aid, with the aim to recognize the weaknesses in this topic to facilitate addressing these areas in the future. Worldwide, 195,000 deaths occur annually from burns [1]. A meta-analysis done by Othman et al. demonstrated that the incidence of burns in the East Mediterranean region ranges from 112 to 518 per 100,000 per year; mortality was reported to be from 0.2 to 5.6 per 100,000 per year, with homes being the commonest location of burn injury ranging from 72 to 94 % [2]. The modern-day household is the most common location where burns occur; inhabitants who smoke cigarettes, the presence of electrical appliances, cooking, water heaters, and the presence of chemical products make this the case. This is evident when examining the location of domestic burns where the kitchen and bathrooms were the most common sites [3, 4]. Several studies have been published reporting the epidemiology of burns in Saudi Arabia with common findings. Scald and flame burns predominated, and homes were the commonest location with children being the most common age group afflicted, most of which were under 5 years. Most of these studies also showed that burns were more common in males. Additionally, mortality was generally low ranging from 4.4 to 9.4 % [5–11]. These findings are consistent with the international data from other countries [2, 12, 13].

The cost of treating burns represents a major burden on governments and healthcare systems. In 2000, the direct cost for care of children with burns in the USA exceeded 211 million dollars [1]. In Germany, a total of 270,000 Euros per patient per year are spent on burn victims [14], and in the UK, it was about £63,157 Pounds [15]. Preventive measures and burn first aid have been proven to reduce morbidity and mortality from burns [16–18]. Studies have shown that certain first aid measures have a beneficial impact on reducing morbidity-related healthcare costs, through limiting tissue damage, leading to a decrease in the need for surgical intervention [16].

With regard to first aid, stopping the burn process, the immediate application of running cold tap water for a time period of 20 min, removing clothing and jewelry, and covering the wound with a sterile dressing would all positively affect the outcome of burns [19–22]. Cold water that has been shown to improve outcome in terms of healing and final cosmetic result should be from 2–15 °C and applied immediately for the same time frame [19]. If the application of water is slightly delayed or if it is applied for less than 20 min, there will still be some beneficial effect based on animal histological section studies but the analgesic advantage of immediate application would be lost [20, 23, 24]. Furthermore, the beneficial effects of cooling the burn wound should not deteriorate the general condition of the patient (like hypothermia in children) [22].

Improper practices of burn first aid can have detrimental consequences. Ice is a common measure used by victims and first responders; ice increases the risk of hypothermia especially in larger surface area burns [19]. A common misconception in many areas is the use of eggs, toothpaste, mud, and other traditional remedies that are harmful, certainly aggravating the injury and creating a more favorable environment for infection, and at best, these remedies are of no benefit [25–27].

Saudi Arabia and other Muslim communities represent a unique setting where burns of specific social nature arise, for example, the weekly Friday mass prayer and the annual pilgrimage in Mecca are regarded as high-risk gatherings for burns [28, 29]. The holy month of Ramadan in which Muslims fast from dawn to sunset has potentiated burns in epileptic patients not taking their medications [30]. Other specific events include the Muslim Eid Alfitr/Aladha where fireworks are used for celebration [31]. In this article, we aimed to assess the general knowledge and practices related to burn first aid implementation together with tendency towards the use of traditional remedies to manage these injuries or to treat them.

## Methods

An Internet-based cross-sectional study with a survey was designed using a 30-part questionnaire submitted over a 4-week period through different social media platforms to assess the knowledge and practices of the Saudi general population with regard to first aid in burn injuries and traditional remedies. The questionnaire included information on socio-economic and demographic variables and retrospective questions on what first aid measures respondents’ use. Participation in the survey was ensured to be anonymous.

The web-based questionnaire was developed and designed using Survey Monkey (SurveyMonkey.com) as the platform. The survey was made in Arabic language to make it easier for the public to read and understand. Then, the results were translated to English using proper accredited translation tools. Survey Monkey is a web-based, flexible, scalable, and secure survey development tool. It was piloted on 15 participants with the clarity of questions, time consumed, and flow of contents utilizing both an electronic actual version and a printed version on different applicant with various demographics including age, sex, education level, and social class. A panel of expert plastic surgeons to ensure proper content reviewed the survey. The questionnaire responses were anonymous. Reviewing the IP address of respondents was performed to prevent duplication.

Data was encoded into Microsoft Excel worksheets and imported to Statistical Package for the Social Sciences (SPSS**®**) software for analysis. Descriptive data was expressed as percentages. Correlations between variables were performed using Pearson Correlation coefficient. Significance of correlations was tested using chi-square test for categorical variables. *P* values <0.05 were considered statistically significant.

## Results

At the end of the assessment period, a total of 2758 individuals responded to the survey. With regard to the demographics of screen population, 41.7 % of respondents were between 19 and 25 years old. Two thirds of them were females. The majority of the respondents were well educated (had a bachelor degree or higher). Most of the sample population (84.9 %) had a monthly income less than 20,000 Saudi Riyals (5333 $). (Data is summarized in Table 1.)

**Table 1:** Demographic characteristics of respondents

Demographic variables	Number	Percentage
Age groups		
15–18 years old	379	13.7
19–25 years old	1149	41.7
26–35 years old	698	25.3
36–45 years old	351	12.7
46 and above	181	6.6
Gender		
Male	942	34.2
Female	1816	65.8
Level of education		
Less than high school	122	4.4
High school	710	25.7
College	210	7.6
University degree—Bachelor’s	1408	51.1
Masters, PhD	300	10.9
None	8	0.3
Nationality		
Saudi	2638	95.6
Non-Saudi	120	4.4
Residence location		
Central	1702	61.7
Western	502	18.2
Northern	171	6.2
Southern	117	4.2
Eastern	266	9.6
Monthly income in Saudi Riyals		
Less than 10,000	1502	54.5
10,000–20,000	840	30.5
21,000–30,000	196	7.1
More than 30,000	220	8.0
Type of residence		
Villa	1823	66.1
Apartment	935	33.9
With children/adolescents/teenagers (under 18) living at home		
Yes	2202	79.8
No	556	20.2
Received information on burn first aid	1178	42.7
Sources of information		
Official courses	523	44.4
Internet	510	43.3
Pamphlets	506	43.0
Television	418	35.5
Newspaper	123	10.4
Websites	62	5.3
Radio	36	3.1

Among the 2758 respondents, 1550 (56.2 %) experienced a burn injury to themselves or a close family member. This subset was directed to an additional set of questions regarding the first aid measures implemented in their experiences (summarized in Table 2).

**Table 2:** Burn first aid implementation

Questions and choices	Number	Percentage
History of exposure to burn injury (self or family member)	1550	–
Directed questions based on history of previous exposure		
Remove clothing or accessories	1118	72.1
Seek primary medical assistance	985	63.5
Wrap injury with clean piece of cloth	526	33.9
Apply water to injured area	990	63.9
Apply cold water to injured area	877	63.0
Apply water for		
Less than 5 min	588	59.4
5–10 min	271	27.4
10–15 min	64	6.5
More than 15 min	57	5.8
Missing data	10	1

Following the burn injury, 1118 (72.1 %) removed clothing and accessories from the area of injury, 526 (33.9 %) wrapped the area with a clean cloth. Water was applied by 990 (63.9 %). Among those who applied water, cold water was applied by 877 (88.6 %). When it came to the duration of water application, 588 (59.4 %) applied water for less than 5 min, 271 (27.4 %) for 5–10 min, 64 (6.5 %) for 10–15 min, and 57 (5.8 %) for more than 15 min, while 10 applicants however did not specify the duration of application. Only 985 (63.5 %) sought medical assistance by visiting an emergency department of plastic surgeon.

Previous background knowledge on burn first aid was present in 1178 respondents (42.7 %). The main source was an official course in 523 (44.4 %), followed by the general internet wedsites in 510 (43.3 %), then pamphlets 506 (43.0 %), television in 418 (35.5 %), newspapers in 123 (10.4 %), specialized websites in 62 (5.3 %), and finally from a radio in 36 (3.1 %).

**Fig. 1:**
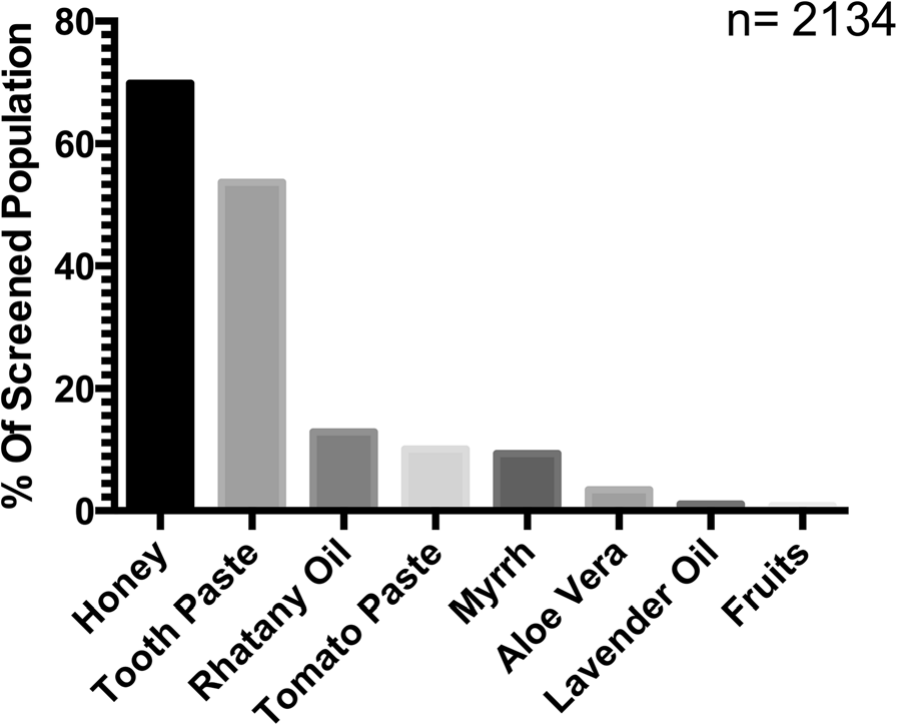
Traditional remedies applied for burn injury. Knowledge and preference towards the application of different home remedies to burn injury among screen population is summarized. Total population screen is shown (*n* = 2134). Data is presented as the percentage of population screened

The knowledge and preference towards the application of home remedies to burn wounds was also assessed (Fig. 1). Among the 2758 people screened, 2134 (77.4 %) knew of and/or used such remedies to treat a burn injury.

Honey was chosen as a remedy to treat burns by 69.9 %, toothpaste was chosen by 53.7 %, while myrrh (*Commiphora myrrha*) was chosen by 9.4 % of respondents. Most of these respondents were females (*r* = 0.87, *P* < 0.001), from younger age group (19–25 years) (*r* = 0.077, *P* < 0.001), from central region (*r* = 0.012, *P* < 0.001), and university graduates (*r* = 0.05, *P* = 0.002). No significant association was found however when assessing the use of home remedies to burn wound with the income level (*P* = 0.67).

Previous knowledge about burn first aid was also correlated to such practices in which 1204 respondents knew of home remedies and did not receive any previous information on burn first aid. There were 915 respondents who still used home remedies despite receiving information on burn first aid but with no statistical significance (*P* = 0.552).

## Discussion

Burn first aid practice represents an essential step of management that dictates the overall outcome of these wounds together with associated financial burden [16-18]. Several studies have been published reporting the proper implementation of first aid steps (summarized in Table 3). In New South Wales, Harvey et al. surveyed 7320 individuals via a telephone-based survey with 82 % of respondents expressing that they would cool a burn with water; however, only 9.4 % would do so for an optimal 20 min; other first aid measures were implemented in less than 1 % [25]. In New York, Taira et al. studied 211 burn victims on their pre-hospital actions, the study showed that 73 % cooled their burns, tap water was used by 39.9 %, ice by 25.2 %, and a cooling blanket by 8.9 %, dressings were applied by 22.2 % only [32]. In Australia, Cuttle et al. studied retrospectively 459 burn victims, with 80.2 % applying cold water but yet again only 12.1 % did so for 20 min or more [29]. In Milas, Turkey 39.6 % applied cold water alone to burn injuries [27]. In Kwa-Zulu Natal, 26 % applied water and only 1 % applied water for 10 min [33]. In our Saudi population sample, water was applied initially by 63.9 % (*n* = 990) of respondents, of which cold water was used by 88.5 %. Most respondents 59.3 % (*n* = 588) applied water for less than 5 min (detailed in Table 2). As shown, Saudi Arabia stands poorly when it comes to proper burn first aid, with the local figures being close to those in Turkey and Kwa-Zulu Natal and far from those in western countries. One important note is that in all countries, the vast majority of the population did not uphold the proper implementation, so that even if water was applied, it was for much less than the optimal duration, thereby limiting the benefit of the recommended measure.

**Table 3:** Burn first aid practices summarized literature

Author	Location	Burn first aid practices
Harvey et al. [25]	Sydney, Australia	82 % of respondents applied water to cool burn wounds 9.4 % applied the water for an optimal 20 min
Taira et al. [32]	New York, USA	73 % of respondents cooled burn wounds 39.9 % tap water was used 25.2 % ice was used Wound wrapping and dressing was applied by 22.2 %
Cuttle et al. [24]	Australia	80.2 % of respondents applied cold water to cool burn wounds 12.1 % applied the water for an optimal 20 min or more
Karaoz B [27]	Milas, Turkey	39.6 % of respondents applied cold water to cool burn wounds
Scheven D. et al. [33]	KwaZulu-Natal, South Africa	26 % of respondents applied cold water to cool burn wounds

Education and literacy level represents an important factor for proper adoption of first aid practice. The reported literacy rate among Saudi population aged 15 and above was 94.4 % (female 91.4 %, male 96.5 %) [34]. The majority of our respondents were university graduates, of which 51.1 % were holding a bachelor degree (detailed in Table 1).

The previous acquisition of knowledge regarding burn first aid was found in 43.6 %, which meant that less than half of respondents had received such information, translating to a poor penetration of awareness campaigns in the community. An upside to this is that the top two sources for the information with a combined percentage of 88.1 % were from an official course or a pamphlet, which usually are revised and correct methods of dissemination.

Knowledge about traditional home remedies applied to burn wounds was high among our screened subjects, in which 77.4 % of respondents knew of and potentially would use them for burn first aid. This figure is substantially higher compared to other populations, which might reflect a rigid trust of their efficacy as what was noticed in other studies [33]. Suleiman AK conducted a cross-sectional study among Saudi population to evaluate the level of awareness and safety of different herbal remedies and showed that 91.1 % of respondents did not ask for an expert (pharmacist, physician) opinion prior to such use while 66.2 % relied on friends’ or relatives’ recommendation. Furthermore, 81.2 % of respondents believed that the use of herbal remedies is harmless [35].

Various international reports have shown, for example, in Ghana, mud, burned snail shell, Gentian Violet paint, and eggs were applied to 75 % of the screened pediatric patients with burn wounds [36]; in Kwa-Zulu Natal, 59.2 % used substances like eggs, butter, and soap [33]; in Milas, Turkey, 51 % applied similar traditional remedies [27], whereas in Brisbane, Australia, only 14 out of 459 subjects (3.1 %) applied such remedies [29]. In Bangladesh, other traditional remedies were reported that ranged from raw eggs, banana trees, mud, toothpaste, onion, raw potato mash to coconut oil, kerosene oil, sesame oil, and juice of “kapila” leaves used mostly in rural population [26].

The use of different remedies might reside in the fact that such wounds are painful if exposed and with the improper knowledge about correct and clear first aid measures such application provides a temporary relief [19, 29].

Specific herbs create quite a controversy; aloe vera, tea-tree oil, and lavender oil have anti-bacterial and anesthetic effects, but their effect, as first aid measures that improve outcome, have shown conflicting data, with a general impression from various studies is that they do no harm but are of no benefit either [19, 37]. For other remedies, deleterious effects might occur. For example, an incident of an anaphylactic reaction after the application of eggs to a burn wound was reported in the USA in an immigrant African family that was luckily treated successfully [37].

Toothpaste as an option to manage burns was chosen by 53.7 % of respondents (*n* = 1147). This was also noticed in other countries like Turkey (1.9 %), UK (4 %), and Australia with documented harmful effects with possibility to exacerbate the initial injury [27, 38, 39].

Pure honey on the other hand might represent an interesting modality, as honey is believed to have abilities to stimulate tissue growth, enhance epithelialization, and minimize scar formation [40].

Our results showed that 69.9 % of the respondents knew of and potentially applied honey as a first aid measurement to their burns. Such practice was also reported with different extent across the world [33, 41–43]. Honey was compared to different clinical modalities like the use of mafenide acetate or silver sulfadiazene. Interestingly enough, the application of honey over burn wounds was shown to have reduced the inflammatory reaction, enhanced wound healing, and better control of infections, and to less likely cause contracture or hypertrophic scars [44, 45]. On the other hand, recent results of a systematic review evaluating the use of honey in comparison with other conventional dressing protocols found serious doubts and may in fact delay the healing potential of partial and full thickness burns compared to early excision and grafting [46].

Overall, when it comes to burn first aid, the use of the scientifically studied and recommended treatments is much more simple, available, inexpensive, and has no potential for harm if applied appropriately. Different cultural remedies that are of no clinical benefits together with potential delay in the wound management by masking the extent of burn injury all should be avoided.

Local current statistics showed 21.6 million registered Saudi Internet users with a net population penetration of 68.5 % [47]. Putting this in mind, we have utilized this mean of communication to conduct our survey. Despite this, several confounding variables might affect the captured population surveyed with their variability and the potential selection bias to certain questions, all of which might act as a potential limitation to this study.

This however might help in assessing the general population level of knowledge about a particular topic in a relatively easy and low cost technique. Also, it might open an era of further focused investigative work related to this field.

## Conclusions

In conclusion, such results represent the fact that burn first aid practices in Saudi Arabia is poor knowing the fact that the majority of the screened respondents were university graduates (51.1 %), with most victims being mismanaged and potentially worsening the injury. The traditional home remedy practice was also high, which further worsens the current status. The results of this study should be taken into serious consideration by the different healthcare agencies responsible to shed light on deficiencies and to start widespread community incentives. The use of the different means of education about the essential practices of burn first aid management can be further implemented in school and university subjects. Implementation of different community health campaigns should target different audiences at various locations like shopping malls and general public gatherings addressing and demonstrating the proper practices related to burn injuries. The need to control and mass educate the population about the harms associated with the improper application of various traditional remedies without seeking medical attention needs to be implemented in an effort to decrease the morbidity and mortality from this grave injury.
